# Therapeutic Targeting of Exportin-1 in Childhood Cancer

**DOI:** 10.3390/cancers13246161

**Published:** 2021-12-07

**Authors:** Basia Galinski, Thomas B. Alexander, Daniel A. Mitchell, Hannah V. Chatwin, Chidiebere Awah, Adam L. Green, Daniel A. Weiser

**Affiliations:** 1 Department of Pediatrics, Albert Einstein College of Medicine, Bronx, NY 10461, USA; bgalinski@uchicago.edu (B.G.); daniel.mitchell@einsteinmed.org (D.A.M.); chidiebere.awah@einsteinmed.org (C.A.); 2 Department of Pediatrics, University of North Carolina, Chapel Hill, NC 27599, USA; talex@email.unc.edu; 3 Morgan Adams Foundation Pediatric Brain Tumor Research Program, University of Colorado School of Medicine, Aurora, CO 80045, USA; hchatwin@luc.edu

**Keywords:** Exportin-1, nuclear export, childhood cancer, selinexor, SINE compounds

## Abstract

**Simple Summary:**

Exportin-1 is a nuclear transport protein that is overexpressed in cancer cells and associated with inferior outcomes across a range of malignancies. Selinexor is a novel FDA-approved inhibitor of Exportin-1 (XPO1). Although significant research has focused on integration of selinexor into the treatment regimens of adult cancers, it is increasingly recognized that XPO1-directed therapy may be effective as part of management of childhood cancers. We therefore summarize the history of, and latest knowledge about, the function and therapeutic inhibition of XPO1 as it relates to childhood cancer pathogenesis and treatment.

**Abstract:**

Overexpression of Exportin-1 (*XPO1*), a key regulator of nuclear-to-cytoplasmic transport, is associated with inferior patient outcomes across a range of adult malignancies. Targeting XPO1 with selinexor has demonstrated promising results in clinical trials, leading to FDA approval of its use for multiple relapsed/refractory cancers. However, XPO1 biology and selinexor sensitivity in childhood cancer is only recently being explored. In this review, we will focus on the differential biology of childhood and adult cancers as it relates to XPO1 and key cargo proteins. We will further explore the current state of pre-clinical and clinical development of XPO1 inhibitors in childhood cancers. Finally, we will outline potentially promising future therapeutic strategies for, as well as potential challenges to, integrating XPO1 inhibition to improve outcomes for children with cancer.

## 1. Introduction

Childhood cancer, affecting more than 15,000 children per year in the United States and over 300,000 per year globally, is the result of tumor and host biology that is different than adult cancers [1]. Childhood cancers tend to arise spontaneously, have varying degrees of hereditary contribution, and are generally not the result of an accumulation of genetic mutations over a lifetime [2]. Therapy for childhood cancer is often multi-modal, including chemotherapy, surgery, radiotherapy, and/or immunotherapy. Five-year overall survival for children with cancer currently exceeds 80% in many high-income countries, yet a tremendous unmet need exists to improve survival and reduce long-term sequelae of treatment. First, we must better identify subsets of patients who have high-risk disease with inferior outcomes and develop improved therapeutic strategies for their resistant disease [3,4,5]. Second, therapy de-intensification may be considered for cancers with favorable clinical and biological features and a relatively high overall survival rate, so that treatment-related morbidity can be minimized [6]. Third, identifying therapeutically targetable oncogenic vulnerabilities may allow for personalized and more effective approaches to care. Recent advances in the understanding of adult malignancies have demonstrated the relevance of Exportin-1 (*XPO1*) overexpression in highly aggressive cancers and the potential for precise therapeutic targeting, yet the overall understanding of this protein in childhood cancer is less well established. In this review, we will focus on clinical implications of overexpression of *XPO1*, including pharmacologic targeting with commercially available XPO1 inhibitors such as selinexor.

## 2. XPO1 Biological Function and Relevance

XPO1, historically referred to as CRM1 (Chromosomal Region Maintenance 1), was initially characterized for its role in mitotic spindle activity and chromosome assembly [7,8]. During development, *XPO1* is ubiquitously expressed, and Xenopus and Drosophila studies demonstrated the requirement for early expression that continues through development; *XPO1* is embryonic lethal if genetically knocked out [9]. Studies in yeast have demonstrated that mutations in *XPO1* lead to a reduction in cytoplasmic microtubules and improper spindle formation [10,11]. Microdeletions of *XPO1* in autism spectrum disorders have also been identified and result in disruption of mitosis leading to apoptosis in neural progenitors [12].

### 2.1. Regulation of Homeostasis by Exportins

Cellular homeostasis depends heavily on the balance of activity and localization of proteins, as together they affect signaling pathways that can either promote cellular survival or cell death. This balance is tightly regulated to maintain healthy cellular conditions, but in cancer, changes to the activity and localization of proteins can promote survival, growth, and metastasis [13,14]. The karyopherin protein family, which is comprised of importins, transportins, and exportins, shuttles cargo (proteins, tRNA, and microRNA) between the nucleus and cytoplasm through the nuclear pore complex [13,15,16,17]. These cargos, sized 30 kDa or greater, are not able to diffuse freely from one compartment to another. They rely on the gradients of high RAN-GTP in the nuclear compartment and high RAN-GDP in the cytoplasmic compartment to facilitate this energy-dependent translocation [18,19,20,21,22]. This gradient, along with RCC1 and RanGAP to catalyze the exchange of nucleotides, creates a cycling of RAN ensuring proper export and import of cargos [23].

XPO1 is one of seven exportins in humans that regulate the unidirectional export of proteins, tRNAs, and microRNAs from the nucleus to the cytoplasm using the nuclear pore complex [24]. Exportins recognize and bind to a leucine rich hydrophobic region, known as a nuclear exportation signal (NES), on cargo (with RNA species using adapter proteins) and, with RAN-GTP in a complex, translocate to the cytoplasm [25,26,27]. The hydrolysis of RAN-GTP to RAN-GDP releases the complex, allowing the cargo to diffuse freely in the cytoplasm, while the exportin returns to the nucleus through the nuclear pore [19]. While certain exportins recognize few proteins or microRNAs exclusively, XPO1 recognizes the nuclear export signals of over 200 proteins and microRNAs. Characterization of the numerous cargos with NES binding motifs was initially established through use of leptomycin B, an antifungal agent that recognizes the NES site of XPO1 and blocks cargos from forming the exportation complex, leading to nuclear accumulation of cargos [8,28]. Current approaches apply in silico modeling to identify and validate XPO1 cargos (http://prodata.swmed.edu/LRNes, accessed on 30 June 2021), many of which function in signaling pathways and cellular regulation, with implications for understanding the pathogenesis of childhood cancer and the effect of pharmacologic inhibition of XPO1 (Table 1). The promiscuity in cargo exportation by XPO1, which is universally expressed across human cells, is consistent with its vital, non-redundant intermediary role in normal homeostatic processes [27,29].

At centrosomes and kinetochores, XPO1 colocalizes with RAN-GTP and cargos with NES that are important for normal cell division [40,41]. Recruitment of XPO1 to these sites is via phosphorylation by the CDK1/Cyclin B complex at the serine 391 site of XPO1, which is a different site than where it binds to various cargo NES [42,43]. One cargo, Survivin, which is upregulated during cellular development and is a member of the inhibitor of apoptosis family, complexes with XPO1 to facilitate normal cellular division through assembly of the chromosome passenger complex during mitosis [44,45,46]. Increased expression of Survivin is also associated with cancer cell survival; specifically, cytoplasmic Survivin is more abundant when XPO1 is overexpressed and promotes chemoresistance through inhibition of apoptosis [47,48,49,50,51]. While XPO1 and its role in regulating mitosis is important, in the context of cancer targeting and treatment; the focus has been on its role as a nuclear export protein and the disruption of downstream signaling pathways.

### 2.2. XPO1 Function in Human Disease

**Figure 1:**
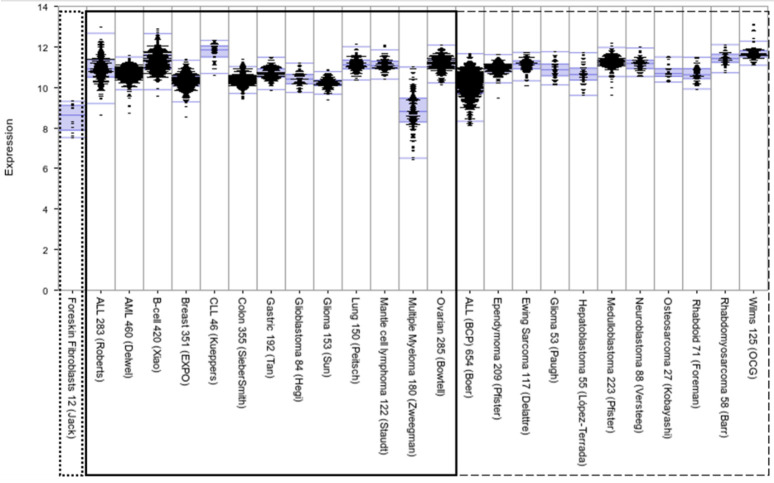
Comparative expression levels of *XPO1* in childhood cancers (**right panel**, dashed box) and select adult malignancies (**left panel**, solid box). *XPO1* is expressed highly across malignancies, including adult and pediatric cancers, reflecting the broad clinical implications of XPO1-focused research. Data are from R2 database (http://r2.amc.nl, accessed on 30 June 2021), including foreskin fibroblast (in the dotted box) as representative of baseline expression in normal tissue.

Across nearly all cancers, including those affecting children, adolescents, and young adults, *XPO1* is expressed (Figure 1) [52,53,54,55,56,57,58], and higher expression has been correlated with inferior outcome. *XPO1* is located on chromosome arm 2p, a region frequently associated with somatic copy number changes in cancer (e.g., gastric cancer, CLL, lymphomas, neuroblastoma, and rhabdomyosarcoma), and a region that includes putative proto-oncogenes *N-MYC*, *REL*, and *BCL11A* [7,59,60,61,62]. This region may be key to understanding how *XPO1* regulation is coopted to promote oncogenesis and to distinguish between sufficiency and necessity of overexpression to promote aggressive disease.

For example, *N-MYC* is amplified in approximately 20% of neuroblastomas, expressed in other pediatric cancers, and associated with more aggressive disease [63,64,65]. It is expressed primarily in neural tissues, in contrast to ubiquitously expressed C-MYC, sharing redundant functions and cross regulation in normal development [66,67,68]. In adult cancers, pharmacologic targeting of XPO1 decreases *C-MYC* levels [69]. Though there is not a clear understanding of what drives *XPO1* overexpression as a primary or secondary event in tumor development, it has been shown that knockdown of the tumor suppressor *TP53* results in increased *XPO1* expression, while knockdown of the proto-oncogene *C-MYC* results in decreased *XPO1* expression [70]. Dysregulation of *TP53* and *C-MYC* could be an initiating event that leads to overexpression of XPO1 [70,71,72,73].

*XPO1* mutations have been identified, particularly in patients with hematologic malignancies [74]. The prevalence ranges from 8% in chronic lymphocytic leukemia (CLL) (primarily D624 mutation) to 25% in primary mediastinal B-cell lymphoma (PMBL) and classical Hodgkin lymphoma (cHL) (primarily E571 mutations). *XPO1* R749 mutations have also been identified in solid tumors [75,76,77]. In limited studies of CLL, *XPO1* mutations correlate with inferior outcome and the need for early aggressive therapeutic intervention [78,79,80,81]. It is unclear if this correlation is due to co-occurrence with *TP53* mutations, or a direct effect of the mutation leading to pathologically increased cargo binding affinity and depletion of regulatory cargos from the nucleus [76]. Leukemia and lymphoma cells with E571K mutations have increased sensitivity to selinexor in vitro, suggesting that upfront screening for mutations may have utility for precision medicine approaches utilizing XPO1-based management strategies [75].

## 3. Development of Selective Inhibitors of Nuclear Export for Cancer Treatment

Selinexor was FDA approved in 2019 as part of combination therapy for relapsed multiple myeloma, an exclusively adult malignancy [82]. It was approved in 2020 as a single agent for adults with relapsed/refractory diffuse large B cell lymphoma (DLBCL) [83]. While adolescents can develop DLBCL, the currently approved indications for selinexor do not directly translate to most pediatric tumors. However, parallel preclinical and early clinical trial efforts have been ongoing, focused on tumor biology related to XPO1 and safety of selinexor for children with cancer.

### 3.1. Preclinical Development of XPO1-Directed Therapeutics for Childhood Cancers

#### 3.1.1. XPO1 Inhibitors as Single Agent Treatment

Many early pharmacologic compounds that inhibit XPO1, such as Ratjadone C (myxobacterial metabolite), KOS-2464 (leptomycin B derivative), N-azolylacrylate analogs, valtrate, and acetoxychavicol acetate have been identified in vitro to bind competitively to the cystine 528 residue on XPO1, leading to nuclear retention of cargos such as TOPO2A and the HIV Rev protein [84,85,86]. These agents were not tested in vivo and safety and tolerability have not been established; therefore there are no data to encourage clinical testing. Other compounds that inhibit XPO1, such as the antifungal agent leptomycin B (elactocin), showed numerous irreversible off-target effects, including severe anorexia and malaise, that limited its clinical advancement [87,88].

Four selective inhibitors of nuclear export (SINE) compounds that reversibly bind XPO1, KPT-185, KPT-251, KPT-330 (selinexor), and SL-801 (felezonexor), have been explored across a range of malignancies. Preclinical evaluation of SINE compounds in specific childhood cancers dates back to at least 2012 with agents demonstrating low single-agent IC_50_ concentrations across various pediatric leukemia subtypes [89,90,91,92], high-grade gliomas [92,93], atypical teratoid/rhabdoid tumor (ATRT) [92], malignant rhabdoid tumor (MRT) [92], sarcoma [92,94], neuroblastoma [30,92], and Wilms tumor [92]. Patient-derived xenograft (PDX) mouse models have shown encouraging anti-tumor activity and survival advantages in treated animals [89,90,91,92]. A study by the Pediatric Preclinical Testing Consortium utilizing pediatric xenografts, including gliomas, leukemias, and sarcomas showed improvement in event-free survival for 29 out of 38 (76%) solid tumor models and 5 out of 8 (63%) leukemia models [92].

Similar to findings in adult malignancies, XPO1 inhibitors in pediatric cancers have been found to induce a G1 cell cycle arrest, leading to a decrease in cells entering S/G2, in leukemia, neuroblastoma, MRT, and ATRT [30,89,90,91,95]. Further, XPO1-directed therapy has been shown to upregulate apoptosis in leukemia, high-grade glioma, ATRT, neuroblastoma, MRT, and sarcoma [30,89,90,91,93,94,95]. In acute leukemia, Etchin et al. demonstrated the effect of KPT-185 on G1 cell cycle arrest and showed that the majority of apoptosis is induced at G1 [89]. These results provide insight into potential combination strategies that would promote a synergistic anti-cancer activity based on convergence of anti-cancer mechanisms.

#### 3.1.2. Selinexor-Based Combination Strategies with Mechanistic Rationale for Use in Pediatrics

The success of the reversible SINE compounds as single agents prompted further investigation of combinatorial strategies to improve efficacy and offset potential dose-related toxicity concerns through lower dosing. The results demonstrating that selinexor-induced apoptosis occurs in the G1 phase of the cell cycle suggests potential for synergy when combined with conventional cytotoxic chemotherapy. Many commonly used chemotherapeutics have their largest effects in the cell cycle phases S, G2, and M. Thus, combining SINE compounds with fludarabine and cytarabine (primarily S phase effects) for acute myeloid leukemia (AML), anthracyclines (primarily S/G2 effects) for soft tissue sarcomas, or topotecan (S/G2 effects) or paclitaxel (M phase effects) for solid tumors are rational approaches for further study [96,97,98,99,100,101,102].

Selinexor has been combined with proteasome inhibition. The most well-studied combination in adults is selinexor in combination with bortezomib and dexamethasone for the treatment of multiple myeloma. Based on these data, the combination of selinexor and proteasome inhibition is being explored in pediatric tumors, including neuroblastoma. Researchers demonstrated synergistic anti-cancer activity and increases in total apoptosis that were mediated by IkB nuclear localization induced by selinexor and diminished IkB degradation with bortezomib; they also identified synergistic functional inhibition of NF-kB transcriptional activity [30].

Combinations of decitabine, a DNA methyltransferase inhibitor, and selinexor have also been investigated. In vitro studies in AML showed no benefit of concurrent treatment, but pre-treatment with decitabine followed by selinexor showed synergy. The findings were recapitulated in a PDX murine model, where pre-treating with decitabine followed by sub-IC_50_ selinexor dosing, lead to a survival advantage over single agent selinexor [103]. Priming with decitabine may increase selinexor tolerability and effectiveness by 30–47% as increasing DNA methylation induces re-expression of tumor suppressor proteins, which are later trafficked by XPO1 [104].

Selinexor for use in children with cancer is also being explored with other novel therapies, including small molecules targeting CDK 4/6 (palbociclib) and Wee1 (AZD1775). In a study focusing on a single pediatric patient-derived cell line from a rare undifferentiated sarcoma, selinexor was effective alone, but combination with palbociclib demonstrated synergy [94]. Other combinations that have been studied include BCL-2 inhibitors [105,106,107,108], PARP inhibitors [109,110], and CAR-T cells [111], with potential implications for many pediatric cancer types, including AML, ALL, anaplastic large cell lymphomas, Ewing sarcoma, neuroblastoma, osteosarcoma and glioblastoma. Immune targeting approaches with pembrolizumab and rituximab, as well as targeting JAK/STAT and tyrosine kinase signaling pathways may be of value. An additional treatment strategy involves the combination of selinexor with radiotherapy, an essential component of pediatric high-grade glioma management, where selinexor improves sensitivity to ionizing radiation and increases anti-tumor activity in adult patients [93,112].

Selinexor has potential to be rationally combined with numerous established and novel therapeutics agents to create critical synergy against pediatric malignancies. The drug has been shown to offset resistance to conventional chemotherapeutics via numerous pathways related to nuclear transport of key cargo, suggesting that patients with relapsed and refractory cancers might benefit from incorporating XPO1 inhibitors into treatment regimens [113,114,115,116]. Continued exploration of combination strategies and timing of drug administration will provide opportunities to define optimal management approaches that can be developed further as clinical trials across a range of cancers that affect children.

#### 3.1.3. Therapeutic Resistance to Selinexor

Specific mechanisms that promote resistance to XPO1 inhibition have not yet been fully elucidated. One group was able to create selinexor-resistant leukemia models through a CRISPR-induced heterozygous mutation in the XPO1 cargo-binding pocket (C528S), rendering XPO1 resistant to degradation in these cells [86]. The unique ability of C528S to induce resistance has only been observed preclinically whereas a clinically described E571K mutation in B-cell lymphoma cell lines has no effect on selinexor efficacy [75,106]. Other studies of resistance mechanisms have focused on possibilities outside of XPO1 mutations. As part of preclinical investigation in hematologic malignancies, one group chronically treated cells with increasing concentrations of selinexor and was able to create a resistant line with upregulated NF-kB activity [117,118]. They found that combination with proteasome inhibitors re-sensitized cells to selinexor, indicating that resistance might be overcome through drug combination regimens [119]. Ongoing work is focused on identifying molecular pathways involved in resistance to XPO1 inhibition, such as increased NF-kB signaling, TGF-β/SMAD pathway, and ESF1 transcription activity of G1/S cell cycle [120,121,122,123].

#### 3.1.4. Toxicity Challenges and Next Generation SINE Compounds

Experiments in PDX models [89,90] and clinical trials in adults and children have identified mild and serious selinexor-induced adverse effects. In adults, the most common noted side effects are nausea, emesis, decreased appetite/weight, confusion, neutropenia, thrombocytopenia, anemia, fatigue, infections, hyponatremia and blurred vision [124,125]. The most common selinexor-related toxicities in children have been electrolyte abnormalities (hyponatremia and hypokalemia), elevated liver function enzymes, nausea, and diarrhea [96,126]. Grade 4 tumor lysis syndrome after selinexor administration has also been seen [126]. Dose-limiting toxicities (DLT) include pancreatitis, cognitive disturbance, and reversible cerebellar toxicity [96,126].

A second-generation XPO1 inhibitor, KPT-8602, has been rationally designed to have increased reversibility and limited blood-brain barrier penetration with the intention to reduce toxicities. This toxicity reduction was clear in murine models using KPT-8602, where mice tolerated increased dosing of KPT-8602 without the anorexia and weight loss that was seen with selinexor dosing [127]. This design would not be appropriate for CNS malignancies [128]. Thus far, KPT-8602 therapeutic activity has been studied preclinically in pediatric leukemias, where IC_50_s were found to be even lower than selinexor [127,129]. Similar to selinexor, KPT-8602 leads to increased apoptosis and nuclear retention of TP53 [129], while causing minimal toxicity to normal hematopoietic precursors [127]. In murine PDX models of ALL and AML, KPT-8602 successfully reduced disease burden and extended survival, with evidence indicating that its anti-leukemic activity and elimination of leukemia-inducing cells are superior to selinexor [127,129]. Similar to selinexor, KPT-8602 has demonstrated synergy with other chemotherapy agents. Dexamethasone and KPT-8602 synergistically increase apoptosis in multiple ALL cell lines. In T-ALL and B-ALL mouse models, combination treatment resulted in increased survival compared to single agents [100]. In addition, some AML cell lines were found to be synergistically vulnerable to KPT-8602 and venetoclax, a BCL2 inhibitor that promotes apoptosis. The combination resulted in greater nuclear TP53, decreased MCL1, and increased apoptosis than with either drug alone; this combination also resulted in greater reduction of leukemic cells in an AML xenograft model [105].

### 3.2. Clinical Development of XPO1-Directed Therapeutics for Childhood Cancers

As has been the case for many agents in development, the early phase selinexor clinical trials technically allowed for enrollment of older children, yet accrual goals have rarely been achieved. In 2015, a clinical trial designed to assess the tolerability and efficacy of selinexor in patients with AML was open to enrolling children, adults, and older adults, but only enrolled patients 44 to 76 years old, with a median age of 61 [130]. In 2019, researchers began a clinical trial to assess the adverse event rate, MTD, and ORR of selinexor and ixazomib combination treatment in patients with sarcomas aged 14 years or older, but the trial was discontinued after 9 months due to failure to enroll any participants [131]. 

Two studies on the use of selinexor in pediatric patients have been completed [96,126]. The first was a breakthrough phase 1 study of 19 patients examining the use of selinexor in a combination with fludarabine and cytarabine in relapsed or refractory AML or mixed phenotype acute leukemia (MPAL) [96]. Based on preclinical research and early clinical studies in adults, researchers believed that the DLTs from selinexor were related to anorexia and weight loss, so they chose to use a chemotherapeutic regimen known to have different toxicity related to myelosuppression [96]. For the first two weeks of treatment, patients received single agent selinexor to evaluate safety and efficacy, followed by fludarabine and cytarabine on days 15–19 [96]. Following the treatment schedule prescribed, severe nausea/vomiting and fatigue were uncommon [96].

The treatment responses were encouraging. On day 15, after only receiving single agent selinexor, three (out of 15) patients experienced complete response with undetectable MRD (<0.1% based upon flow cytometric analysis) [96]. After completing one cycle of combination treatment, 7/15 patients (47%) reached complete response or complete response with incomplete count recovery, with five of these patients MRD negative [96]. Six of those seven patients were in remission at a median time of 9.5 months from enrollment [96]. One of these six patients with durable response had AML with t (6;9) translocation, which has been shown to have a high risk of relapse and poor outcomes [96,132,133]. This translocation forms a chimeric *DEK-NUP214* gene, which creates an altered nucleoporin fusion protein known to interact tightly with XPO1 [134]. There have been other studies evaluating the response of known oncogenic mutations to XPO1 inhibition, but to our knowledge, this is the first pediatric mutation that may predict therapeutic response to selinexor [76,77,96,135].

Another clinical trial evaluated single agent selinexor in relapsed/refractory AML, ALL, BC-CML, and MPAL [126]. This trial found that single agent selinexor was well tolerated in children apart from two DLTs of pancreatitis and cognitive disturbance [126]. The outcomes of this trial included a ORR of 12.5%, with one patient experiencing complete remission with incomplete platelet recovery and 11/16 patients having a clinical benefit defined by reduction in transfusions, clearance of peripheral blasts, and decreased pain [126]. In addition to these two completed trials, there are five active clinical trials using selinexor open to enrolling pediatric patients, with two in the process of recruiting (Table 2, based on clinicaltrials.gov, accessed on 30 June 2021) [136]. Phase 1 study results from the first trial of selinexor in recurrent pediatric brain and solid tumors (NCT02323880) were recently presented [137]. Primary toxicities in this population were hematological and gastrointestinal, with DLTs on a weekly treatment schedule of thrombocytopenia (2 subjects) and seizure (1 CNS tumor patient). The recommended phase 2 dose was established as 35 mg/m^2^/dose weekly. Treatment response results are still pending from this trial.

## 4. Future Potential of XPO1 Inhibition in Pediatric Oncology

### 4.1. Precision Medicine Using XPO1 Inhibition

An ideal biomarker facilitates upfront risk stratification and accurate prediction of which patients are most likely to benefit from a specific therapy. Overexpression of *XPO1* portends a poor prognosis in some diseases, which implies XPO1 activity drives or is the result of aggressive disease. This has been an attractive area of investigation in the search for a predictive biomarker of selinexor effectiveness [53,55,56,57,80,117,138]. However, *XPO1* transcript expression levels have not been generally predictive of response to targeted inhibition. Specifically in neuroblastoma, XPO1 protein expression and RNA expression do not correlate with sensitivity to selinexor [30]. However, recent efforts have explored XPO1 cargo localization and function, such as those listed in Table 1, as predictors of response to XPO1 directed therapy [121,139]. For example, in gastric cancer, the presence of functional TP53 in the nuclear compartment seems to be crucial to promoting TP53-dependent apoptosis with XPO1/proteasome co-inhibition [140]. The anti-apoptotic protein Survivin could be a reasonable biomarker of sensitivity to XPO1 inhibition. In pediatric cancers, inferior outcome correlates with high levels of Survivin expression, as it functions as an inhibitor of apoptotic signaling [141,142]. Nuclear accumulation of Survivin blocks STAT3 activation, leads to its own degradation, and therefore promotes apoptosis [49,143]. Additional research is focused on therapeutic predictors by identifying molecular pathways involved in resistance to XPO1 inhibition [120,121,122,123]. Development of biomarkers predictive of selinexor response will be key to future application of selinexor in children with cancer.

### 4.2. New Translational Opportunities

Neuroblastoma, osteosarcoma, and high-grade gliomas (HGG) are particularly difficult to treat cancers that affect children, and integration of XPO1 inhibition into therapy has potential to transform management. In neuroblastoma, multimodal therapy is highly toxic and results in a cure rate for those with high-risk disease of approximately 50%, with substantial treatment-associated morbidity in survivors. Numerous publicly available gene expression datasets support the recently published findings from a proteomics screen in neuroblastoma that XPO1 is most highly expressed in patients with inferior outcome [30]. The numerous cargos transported by XPO1, including many affected by combination treatment and implicated in augmenting selinexor activity, reinforces current understand that no single agent or directed therapy can fully undermine the propensity of a cancer cell to survive. Given its broad sensitivity across neuroblastoma cell lines, and its safety when used in children, selinexor is a unique agent that may introduce novel oncogenic vulnerabilities, in particular once there is an improved mechanistic understanding of the role of XPO1 in cancer development and aggressiveness.

Similar to most malignant tissues, osteosarcoma shows increased XPO1 protein expression compared with normal tissues, and its expression is associated with worse prognosis, unrelated to important clinical features such as presence of metastatic disease [58]. Selinexor has shown activity in preclinical testing of osteosarcoma through different mechanisms. XPO1 inhibition blocks nuclear export of CDKN1B, an oncoprotein, leading to growth arrest in vitro [33]. Therefore a rational combination of selinexor with Wee1 inhibition was studied and showed greater inhibition of cell proliferation than either drug alone, with Wee1 inhibition of CDK2 activity and selinexor-induced stabilization of CDKN1B in the nucleus promoting G2/M arrest [33]. In osteosarcoma cell lines, XPO1 inhibition decreases hypoxia inducible factor, a key transcriptional regulator of tumor growth and therapy (including radiotherapy) resistance, emphasizing the potential role of selinexor as a radiation sensitizer [144]. In separate preclinical work in osteosarcoma, neuroblastoma, and other malignancies, selinexor was shown to act partially through inhibition of NF-kB. Resistance to selinexor through high NF-kB expression can be overcome with proteasome inhibition, again pointing to this combination as a rational therapeutic approach similar to what is used for multiple myeloma [119].

HGGs, which include multiple subtypes, such as diffuse midline gliomas, lack effective treatment options and are nearly uniformly fatal [145]. These tumors have been of high interest for XPO1 inhibition due to selinexor’s favorable blood-brain barrier penetrance and preclinical activity in patient-derived models of adult and pediatric HGG [93]. A phase 2 trial of selinexor in recurrent adult HGG showed a promising six-cycle progression-free survival rate and some objective responses [146]. As reviewed above, a phase 1 trial of selinexor in recurrent pediatric brain and solid tumors, with a special focus on HGG, has presented initial results and is ongoing. Clinical combination use with radiation and proteasome inhibition is of interest as well, particularly given promising preclinical data and the ability of both selinexor and proteasome inhibitors to target the NF-kB pathway, which may be a key activating pathway in pediatric HGG. NF-κB targeting by selinexor in pediatric HGG appears to be via inducing NGFR expression and increasing nuclear presence of IkB-a [147].

One current approach to defining response prediction is genome-scale CRISPR-Cas9a-mediated knock out screens, which was recently applied to assess genes and pathways associated with HGG response and resistance to etoposide and temozolomide. This can be readily applied as part of testing of selinexor combination strategies across all childhood cancers [148]. A similar functional screening assay led to development of a cancer dependency map in *MYCN*-amplified neuroblastoma that identified a novel protein, Nuclear Transport Factor 2 Like Export Factor 1 (NXT1, p15), that is essential for mRNA nuclear export and cellular integrity [149]. Targeting of NXT1, like XPO1, promotes cell lethality and reinforces the importance of additional research focused on nuclear export machinery in childhood cancer.

## 5. Conclusions

*XPO1* overexpression is nearly universally associated with inferior outcome in cancer. Development of XPO1-directed treatment strategies has been brisk, though pediatric-focused studies have lagged. Our review of the current knowledge about XPO1 and XPO1 inhibition in childhood cancer offers an enthusiastic outlook about the potential for integrating XPO1-directed therapy as part of combination treatment to improve outcomes for children with highly aggressive malignancies. Selinexor, the lead clinical-grade XPO1 inhibitor, holds promise to improve treatment efficacy while minimizing toxic effects of conventional therapy. Improved understanding of regulated cargos, functionally related pathways, mechanistic implications, and biomarkers of efficacy will help guide optimal clinical incorporation of XPO1 inhibition.

## Figures and Tables

**Table 1:** Select XPO1 cargos implicated in childhood cancer pathogenesis.

Cargo	Normal Function	Result of XPO1 Inhibition	Citation
IkB	NF-kB transcription factor signaling	Inhibition of cell survival-promoting NF-kB transcription factor activity	[30]
Survivin (BIRC5)	Inhibition of apoptosis	Degraded to release inhibition of apoptotic pathway	[31]
CDKN1A (p21)	Cell cycle kinase	Halts cell cycle progression, leading to cell cycle arrest	[32]
CDKN1B (p27)	Cell cycle inhibitor	Halts cell cycle progression, leading to cell cycle arrest	[33]
p53	DNA damage recognition	TP53-dependent apoptosis proceeds	[34]
pRB	Cell cycle regulator	Halts cell cycle progression, leading to cell cycle arrest	[35]
FOXO1	Transcription factor-Differentiation	Promotes sensitivity to selinexor in cisplatin-based combination	[36]
pMAPK	MAPK/Developmental signaling processes	Promotes apoptosis by decreasing pro-survival ERK pathways	[37]
APC	β-catenin signaling	Reduction of β-catenin levels by binding and leading to destruction; halts stemness-inducing Wnt signaling	[38,39]

**Table 2:** Overview of clinical trials involving selinexor in childhood cancer.

Diseases	Treatment(s)	Status of Study	Study Phase	Age Range	# of Pts	Outcome Measure	NCT Number
Malignant Glioma, Recurrent or Refractory Solid Tumors	Selinexor	Recruiting	I	12 mo to 21 yo	68	Frequency of DLT, AE Rate, Antitumor Effect of Selinexor, 6-mo PFS	NCT02323880 https://clinicaltrials.gov/ct2/show/NCT02323880?term=selinexor&type=Intr&age=0&draw=2&rank=1, accessed on 30 June 2021
Relapsed or Refractory Childhood ALL, AML, Mixed Lineage Leukemia, Biphenotypic Leukemia, CML in Blast Crisis	Selinexor	Active, not recruiting	I	12 mo to 21 yo	16	Toxicity Profile, MTD, ORR	NCT02091245 https://clinicaltrials.gov/ct2/show/NCT02091245?term=selinexor&age=0&draw=1&rank=2, accessed on 30 June 2021
Dedifferentiated Liposarcoma	Selinexor or Placebo (Double-Blinded Study)	Active, not recruiting	II–III	12 yo and older	342	PFS of patients receiving 60mg of Selinexor vs. Placebo	NCT02606461 https://clinicaltrials.gov/ct2/show/NCT02606461?term=selinexor&age=0&draw=1, accessed on 30 June 2021
AML, de Novo MDS, MDS, Secondary AML, Secondary MDS	Selinexor after allogeneic stem cell transplant	Completed	I	Child, adult, older adult	12	MTD, DLT, 2-yr PFS, incidence of GVHD, incidence of AE, incidence of non-relapse mortality, assess lymphoid and myeloid chimerism, Overall Survival	NCT02485535 https://clinicaltrials.gov/ct2/show/NCT02485535?term=selinexor&age=0&draw=1, accessed on 30 June 2021
Malignant Glioma, Glioblastoma, Diffuse Midline Glioma/ Intrinsic Pontine Glioma, Anaplastic Astrocytoma	Selinexor and Radiation Therapy	Not Yet Recruiting	I–II	12 mo to 21 yo	36	MTD, ORR, Event free survival, Overall survival	NCT05099003 https://clinicaltrials.gov/ct2/show/NCT05099003?term=selinexor&age=0&draw=2&rank=4, accessed on 30 June 2021
Relapsed and Refractory Aggressive B-Cell Lymphoma	Selinexor in combination with Rituximab, Gemcitabine, Dexamethasone, and Cisplatin vs. 2 other experimental treatment arms vs. active comparator arm	Recruiting	II	16 to 65 yo	320	ORR, AE Rate, Transplantation rate, Stem cell collection rate, Event free survival, Survival	NCT02436707 https://clinicaltrials.gov/ct2/show/NCT02436707?term=selinexor&age=0&draw=2, accessed on 30 June 2021
Relapsed or Refractory AML, Relapsed or Refractory Acute Leukemia of Ambiguous Lineage	Selinexor and Venetoclax with and without chemotherapy	Recruiting	I–II	Up to 30 yo	42	RP2D, Hematologic and Non-Hematologic DLT, CR, Survival	NCT04898894 https://clinicaltrials.gov/ct2/show/NCT04898894?term=KPT330&age=0&draw=2, accessed on 30 June 2021
Refractory or Relapsed AML	Selinexor in combination with Fludarabine, Cytarabine, and Methotrexate/Hydrocortisone/Cytarabine	Terminated (due to slow enrollment)	I–II	Up to 24 yo	37	ORR, CRR, CR with Incomplete Count Recovery	NCT03071276 https://clinicaltrials.gov/ct2/show/NCT03071276?term=selinexor&age=0&draw=1, accessed on 30 June 2021
AML, ALL, MDS, Mixed Phenotype Acute Leukemia	Selinexor in combination with Fludarabine, Cytarabine, and Methotrexate/Hydrocortisone/Cytarabine	Completed	I–II	Up to 24 yo	19	MTD, DLT, Maximum Plasma Concentration, CRR, Overall Response Rate, AUC of Selinexor	NCT02212561 https://clinicaltrials.gov/ct2/show/NCT02212561?term=selinexor&age=0&draw=1&rank=3, accessed on 30 June 2021
Liposarcoma, Malignant Peripheral Nerve Sheath Tumors, Alveolar Soft Part Sarcoma, Ewing Sarcoma, Sarcoma	Selinexor and Ixazomib combination	Withdrawn	II	14 yo and older	0	MTD, AE Rate, ORR	NCT03880123 https://clinicaltrials.gov/ct2/show/NCT03880123?term=selinexor&age=0&draw=1, accessed on 30 June 2021

mo = months old, yo = years old, RP2D = Recommended Phase 2 Dose, DLT = Dose-Limiting Toxicity, %DLT = % of patients experiencing a dose-limiting toxicity at least possibly attributable to Selinexor, PFS = Progression Free Survival, ALL = Acute, Lymphoblastic Leukemia, AML = Acute Myelogenous Leukemia, CML = Chronic Myelogenous Leukemia, MDS = Myelodysplastic Syndrome, ORR = Objective Response Rate, CRR = Complete Response Rate, MTD = Maximum Tolerated Dose, AUC = Area Under the Curve, AE = Adverse Event, GVHD = Graft vs. Host Disease.
